# Mapping of foot-and-mouth disease virus antigenic sites recognized by single-domain antibodies reveals different 146S particle specific sites and particle flexibility

**DOI:** 10.3389/fvets.2022.1040802

**Published:** 2023-01-09

**Authors:** Michiel M. Harmsen, Haozhou Li, Shiqi Sun, Wim H. M. van der Poel, Aldo Dekker

**Affiliations:** ^1^Wageningen Bioveterinary Research, Wageningen University & Research, Lelystad, Netherlands; ^2^Laboratory of Virology, Wageningen University and Research, Wageningen, Netherlands; ^3^State Key Laboratory of Veterinary Etiological Biology, National Foot and Mouth Disease Reference Laboratory, Lanzhou Veterinary Research Institute, Chinese Academy of Agricultural Sciences, Lanzhou, China

**Keywords:** neutralizing antibody, epitope, XL-MS, nanobody, VHH, ELISA, foot-and-mouth disease virus (FMDV)

## Abstract

Vaccination with intact (146S) foot-and-mouth disease virus (FMDV) particles is used to control FMD. However, 146S particles easily dissociate into stable pentameric 12S particles which are less immunogenic. We earlier isolated several single-domain antibody fragments (VHHs) that specifically bind either 146S or 12S particles. These particle-specific VHHs are excellent tools for vaccine quality control. In this study we mapped the antigenic sites recognized by these VHHs by competition ELISAs, virus neutralization, and trypsin sensitivity of epitopes. We included two previously described monoclonal antibodies (mAbs) that are either 12S specific (mAb 13A6) or 146S specific (mAb 9). Although both are 12S specific, the VHH M3F and mAb 13A6 were found to bind independent antigenic sites. M3F recognized a non-neutralizing and trypsin insensitive site whereas mAb 13A6 recognized the trypsin sensitive VP2 N-terminus. The Asia1 146S-specific site was trypsin sensitive, neutralizing and also recognized by the VHH M8F, suggesting it involves the VP1 GH-loop. The type A 146S-specific VHHs recognized two independent antigenic sites that are both also neutralizing but trypsin insensitive. The major site was further mapped by cross-linking mass spectrometry (XL-MS) of two broadly strain reactive 146S-specific VHHs complexed to FMDV. The epitopes were located close to the 2-fold and 3-fold symmetry axes of the icosahedral virus 3D structure, mainly on VP2 and VP3, overlapping the earlier identified mAb 9 site. Since the epitopes were located on a single 12S pentamer, the 146S specificity cannot be explained by the epitope being split due to 12S pentamer dissociation. In an earlier study the cryo-EM structure of the 146S-specific VHH M170 complexed to type O FMDV was resolved. The 146S specificity was reported to be caused by an altered conformation of this epitope in 12S and 146S particles. This mechanism probably also explains the 146S-specific binding by the two type A VHHs mapped by XL-MS since their epitopes overlapped with the epitope recognized by M170. Surprisingly, residues internal in the 146S quaternary structure were also cross-linked to VHH. This probably reflects particle flexibility in solution. Molecular studies of virus-antibody interactions help to further optimize vaccines and improve their quality control.

## Introduction

Foot-and-mouth disease (FMD) affects cloven-hoofed animals, causing vesicular lesions at the feet, mouth and udders of lactating animals. The direct economic losses are high, due to loss of milk-production, growth and draft power. Indirect costs due to loss in trade and other restrictions also impact countries where the disease occurs (1). The causative agent, FMD virus (FMDV), belongs to the genus *Aphthovirus* within the *Picornaviridae* family. FMD is mostly controlled by vaccination. The 7 serotypes of FMDV (O, A, C, Asia1, SAT1, SAT2, and SAT3) by definition lack cross-protection, but within serotypes cross-protection can also be limited. FMD vaccine is mainly produced as inactivated authentic virus capsids adjuvanted in an oil emulsion. However, many novel vaccines are being developed based on virus-like particles (VLPs) that lack the RNA genome, similar to natural empty capsids that sediment in sucrose gradients at 75S (2, 3). For an adequate immune response it is essential that the capsids represent intact (146S) virions. When capsids are heated or kept at a low pH, they disintegrate into stable 12S pentamers that have strongly reduced immunogenicity (4, 5). A monoclonal antibody (mAb 9) was earlier shown to be suitable for FMD vaccine quality control due to its specificity for 146S particles in ELISA (6). This mAb 9 showed high specificity for strain A10/HOL/1/42. We later isolated single-domain antibodies (VHHs) that recognize 146S or 12S particles which can be used in vaccine quality control. The 146S particle specific VHHs M170F and M332F are strictly serotype specific and recognize particular serotype O or Asia1 FMDV strains, respectively, while the 12S particle specific VHH M3F is broadly reactive to many FMDV strains of serotypes O, A, C, and Asia1, although it did not bind SAT2 (5, 7). We have recently isolated 10 FMDV serotype A specific VHHs that consistently showed high specificity for 146S particles (8). Two of these VHHs, M691F and M702F, demonstrated remarkable broad strain specificity when compared to other 146S-specific VHHs. Remarkably, M691F did not recognize 75S particles or VLPs while M702F did bind such empty capsids. Thus, M691F was strictly 146S specific while M702F was specific for both full and empty intact capsids. Since the binding to empty capsids is often unknown we refer to both such specificities as 146S specific in this study. Thus, 146S specificity implies inefficient binding of 12S particles as compared to 146S. Here we study the epitope specificity of the serotype A and Asia1 146S-specific VHHs.

The icosahedral FMDV capsid comprises 60 copies of four capsid proteins, viral protein (VP)1 to VP4. One copy of each VP associates into a protomer, five protomers associate to a pentameric (12S) structure arranged around a 5-fold symmetry axis, and 12 pentamers form the full capsid. The capsid outer surface is formed by VP1, VP2 and VP3 while VP4 is located internally. Upon capsid dissociation VP4 dissociates from 12S particles. The association of 12S pentamers in a 146S particle results in additional 2-fold and 3-fold symmetry axes. FMDV targets host cell receptors, including integrins and heparan sulfate. Protective antibodies can block such virus-receptor interaction to neutralize the virus but other mechanisms also exist (9, 10). Based on sequence analysis of mAb resistant (MAR) viruses, five neutralizing sites have been described for serotype O. Site 1, located at the GH loop and carboxy terminus of VP1, includes VP1–138, 144, 148, 154, and 208. The critical residues of site 2 are VP2–70, 71, 72, 73, 75, 77, 78, 131, and 188. The residues in site 3 include VP1–43, 44, and 45 at the BC loop of VP1 near the 5-fold axis. Site 4 is located at VP3 (VP3–56 and 58), and site 5 has critical residue 149 at the VP1 GH-loop (11–16). These sites identified in serotype O are mostly similar in serotypes A, C (9, 17–21) and Asia1 (22). Antibody competition experiments confirmed such grouping into 5 sites (9), although sites 2 and 4 were found to interact (15). The site recognized by 146S-specific mAb 9 was critically dependent on mutation of VP3-70 although VP3-139 could also be involved (21). Site 1 is a linear epitope located on the VP1 GH-loop, which contains the RGD motif that interacts with integrins (23), whereas all further sites are conformational. Antibody binding to site 1 is sensitive to trypsin treatment of the virions, which is known to cleave the GH loop (23). In addition to these neutralizing sites, mAbs that bind to non-neutralizing sites were identified, including mAb 13A6, which are broadly reactive against FMDV strains from all 7 serotypes and binds a linear peptide representing the N-terminal 15 amino acids of VP2 that is sensitive to trypsin treatment (24, 25).

A set of VHHs binding to serotype O, including VHHs M3F, M8F, M23F, and M220F were earlier mapped into four separate epitope bins indicated by roman numerals I to IV (26). Most of these four sites could not be linked to the earlier described sites 1 to 5 identified by MAR mutant analysis, although M8F binds a linear epitope on the VP1 GH-loop, similar to site 1 binding mAbs. Site III is specific for serotype O whereas sites I, II and IV are present on other serotypes as well (Table 1). M170F competed with VHHs mapped to sites I and III. The epitopes recognized by M8 and M170 were recently determined based on the cryogenic electron microscopy (cryo-EM) structure of these VHHs complexed to FMDV (10). We refer to these VHHs without the suffix F since they were produced in Escherichia coli without the long hinge region, which is present in our yeast-produced VHHs (27). M8 was found to bind predominantly the VP1 GH-loop, in addition to some VP3 residues, while M170 bound mainly to a region on VP3 close to the 3-fold axis. It was proposed that the 146S-specific binding of M170 relies on binding to VP3 residues which assume a different structure in 12S particles as compared to 146S particles, most importantly D71 and V73 in the BC loop and E131 and K134 in the EF loop. Alternatively, it was suggested that 146S specificity could also rely on antibody binding to an epitope that is present on two adjacent pentamers, on both sides of the 2-fold axis, that is separated into two halves upon dissociation into 12S particles (6), although firm proof for this hypothesis is lacking.

**Table 1:** FMDV serotype, strain, and particle specificity of previously-isolated VHHs used.

**VHH^a^**	**FMDV serotype specificity**	**Binding in ELISA to FMDV strains** ^**b**^			**FMDV particle specificity^c^**	**Serotype O antigenic site^d^**	**References**
		**A/TUR**	**A24Cru**	**Asia1 Shamir**			
M8F	O, A, Asia1, C	Y	Y	Y	12S and 146S	I	(26)
M3F	O, A, Asia1, C	Y	Y	Y	12S	II	(26)
M23F	O	N	N	N	12S and 146S	III	(26)
M220F	O, A, Asia1, C	Y	Y	Y	12S and 146S	IV	(26)
M663F	O, A, Asia1, C	Y	Y	Y	12S	ND^e^	(8)
M680F	O, A, Asia1, C	Y	Y	Y	12S	ND	(8)
M665F	O, A, Asia1	Y	Y	Y	12S	ND	(8)
M684F	O, A, Asia1, C	Y	Y	Y	12S	ND	(8)
M675F	A	Y	Y	N	12S and 146S	ND	(8)
M643F	A	Y	N	N	(12S and) 146S	ND	(8)
M652F	A, C	Y	Y	N	(12S and) 146S	ND	(8)
M659F	A	Y	N	N	146S	ND	(8)
M702F	A	Y	Y	N	146S	ND	(8)
M691F	A	Y	Y	N	146S	ND	(8)
M703F	A	Y	Y	N	146S	ND	(8)
M669F	A	Y	N	N	146S	ND	(8)
M676F	A	Y	N	N	146S	ND	(8)
M677F	A	Y	N	N	146S	ND	(8)
M678F	A	Y	N	N	146S	ND	(8)
M686F	A	Y	N	N	146S	ND	(8)
M688F	A	Y	N	N	146S	ND	(8)
M661F	A, Asia1	Y	Y	Y	(12S and) 146S	ND	(8)
M651F	A	Y	Y	N	(12S and) 146S	ND	(8)
M679F	A	Y	N	N	12S and 146S	ND	(8)
M326F	A	N	Y	N	12S and 146S	ND	(8)
M655F	A	N	Y	N	12S and 146S	ND	(8)
M662F	A	N	Y	N	12S and 146S	ND	(8)
M332F	Asia1	N	N	Y	146S	ND	(7, 8)
M658F	Asia1	N	N	Y	146S	ND	(8)
M685F	Asia1	N	N	Y	12S and 146S	ND	(8)
M98F	Asia1	N	N	Y	12S and 146S	ND	(7, 8)

^a^The 14 VHHs of the 7 CDR3 groups comprising the 12 VHHs that bind specifically to 146S of serotype A or Asia 1 strains are color-coded by their CDR3 group, as done earlier (8). Thus, VHHs with the same color belong to the same CDR3 group while VHHs in black all belong to different CDR3 groups.

^b^The A450 values in ELISA obtained earlier (8) were used to determine binding to the indicated FMDV strains, taking A450 = 0.5 as cutoff for binding (Y) or no binding (N).

^c^FMDV particle specificity was determined earlier (8). An EC ratio in ELISA that was > 10 times higher using 146S as compared to 12S was considered 146S-specific binding. Some VHHs showed 146S specificity only using particular strains and are therefore indicated as (12S and) 146S.

^d^Sites I to IV as initially identified in serotype O FMDV (26).

^e^ND, not determined.

In this study we used different approaches to map the antigenic sites recognized by especially the serotype A 146S-specific VHHs earlier isolated, but also the 12S-specific VHH M3F and Asia1 146S-specific VHHs M332F and M658F. These VHHs were analyzed in competition ELISAs, virus neutralization tests and binding to trypsin-treated FMDV antigen. We further mapped the antigenic sites of two type A broadly strain reactive and 146S-specific VHHs, M691F, and M702F (8), using cross-linking mass spectrometry (XL-MS).

## Materials and methods

### Viruses and viral antigens

Production of FMDV antigens of strains A/TUR/14/98 (A/TUR), A24/Cruzeiro/BRA/55 (A24Cru), A10/HOL/1/42, O1/BFS1860/UK/67 and Asia1/Shamir/ISR/89 (Asia1 Shamir) was done as described earlier (8). Briefly, FMDV was amplified in BHK-21 cells grown in suspension in industrial-size bioreactors or 850-cm^2^ roller bottles. FMDV present in the clarified supernatant was inactivated with 10 mM binary ethylenimine and concentrated using polyethylene glycol-6000 precipitation, resulting in crude antigen. Purification of 146S particles by sucrose density gradient (SDG) was done as described earlier (8). Trypsin-treated antigen (TTA) was prepared as described previously (23). A peptide representing the 22 N-terminal amino acid residues of VP2 (DKKTEETTLLEDRILTTRNGHT) derived from O1/Manisa/TUR/69 (Genbank acc. no. AY593823) appended with a C-terminal cysteine residue for conjugation to BSA (VP2-22-BSA) was produced by GenScript Corporation (Piscataway, NJ, USA).

### MAbs and VHHs

The 146S specific mAb 22.9 (6) isolated at our institute against strain A10/HOL/1/42 was called mAb 3.9 in a previous publication, and binds the same antigenic site as mAb 3.32 (21). We refer to them as mAb 9 and mAb 32 in this paper. The non-neutralizing mAb 13A6 was earlier isolated against strain SAT1/ZIM/89 (24). The origin of the VHHs used and their FMDV specificity are shown in Table 1. They were produced in baker's yeast using plasmid pRL188, purified by immobilized-metal affinity chromatography and biotinylated as described recently (27). VHHs produced in yeast using pRL188 are indicated by the suffix F. Only the VHH M655F was found to be N-glycosylated (8). It was deglycosylated by treatment with endoglycosidase H (Roche Applied Science, Mannheim, Germany) according to the manufacturer's instructions.

### Virus neutralization test

VHH concentrations required for neutralization of FMDV A/TUR, A24Cru and Asia1 Shamir were determined as described previously (26) using 100 (30-300) tissue culture infective doses required to infect 50% of the wells (TCID_50_) incubated with duplicate serial two-fold dilution series of VHHs for 1 h. The non-neutralized virus was then detected by adding IBRS-2 cells and 2 days later the plates were read macroscopically after staining the monolayers with amido black. VHH neutralization was calculated using the Spearman-Kärber method (28, 29) and expressed as VHH concentration in the VHH/virus mixture which neutralized an estimated 100 TCID_50_ of virus at the 50% end-point (VNT_50_). Most VHH dilution series started at 1 mg/ml. However, the M220F VHH dilution series started at 0.05 mg/ml since it was produced in baker's yeast at low level.

### ELISA for evaluation of VHH binding to FMDV antigens

The procedures for ELISAs have been described (7, 27). High binding 96-well polystyrene plates (Greiner, Solingen, Germany) were coated with 100 μl/well of 0.5 μg/ml unlabeled VHH in 50 mM NaHCO_3_ buffer (pH 9.6) overnight at 4°C. Plates were incubated at room temperature (RT) with 100 μl/well of a 2-fold dilution series over 12 wells starting at 1 μg/ml 146S of suitable FMDV antigens. Bound FMDV antigens were detected by subsequent sequential incubation with 100 μl/well 0.25 μg/ml of biotinylated VHH and 0.5 μg/ml of a streptavidin horse radish peroxidase (HRP) conjugate (Jackson ImmunoResearch Laboratories Inc., USA). Bound HRP was detected by staining with 3,3',5,5' tetramethylbenzidine. The color reaction was stopped by addition of 0.5 M sulfuric acid (50 μl per well) and the absorbance at 450 nm (A450) was measured using a Multiskan Ascent spectrophotometer (Thermo Labsystems, Finland). A four-parameter logistic curve was fitted to absorbance and FMDV concentrations using the SOFTmax Pro 2.2.1 program (Molecular Devices) and was used to interpolate the Effective Concentration (EC) resulting in a specific A450 value for each VHH or antibody. VHHs or antibodies that did not reach the A450 value defining the EC value were given an EC value of the highest FMDV concentration used (1 μg/ml).

Binding to a BSA-conjugated VP2 peptide was analyzed by coating ELISA plates with 4 μg/ml VP2-22-BSA that were subsequently incubated with 0.5 μg/ml biotinylated VHH or mAb and streptavidin-HRP conjugate as described above. Controls included plates coated with 4 μg/ml BSA or 2.5 μg/ml of crude FMDV antigens of strains A/TUR, A24Cru or Asia1 Shamir.

### ELISA for VHH binning

The ability of VHHs or mAbs to bind independent antigenic sites of FMDV was studied by blocking/competition double-antibody sandwich (DAS) ELISA using biotinylated VHHs and mAb 9 or mAb 32. ELISAs were performed using 0.5 μg/ml unlabeled VHH for coating and subsequent capture of crude FMDV antigens (1 μg/ml 146S), that contained about 20% 12S particles, in addition to 146S. The same VHH as used for coating was used in the next step as biotinylated VHH. For mAb 9 and mAb 32, plates were coated with unlabeled VHH M691F. Initially the optimal concentration of biotinylated VHH or mAb for competition was determined by titration of biotinylated VHH or mAb without competition, as described above for determining EC values. A biotinylated VHH or mAb concentration was selected that provided about 80% of the maximal absorbance value observed with the highest VHH or mAb concentration analyzed. In the final experiment, plates containing VHH-captured FMDV were first incubated with the unlabeled VHH or mAb (5 μg/ml) in 90 μl/well for 30 min (blocking step). Then, without washing plates, 10 μl biotinylated VHH or mAb in the predetermined concentration was added and incubated for another 30 min (competition step). A control without antigen and a control without biotinylated VHH or mAb were included. Bound biotinylated VHH was detected by incubation with 0.5 μg/ml streptavidin-HRP conjugate. Bound mAbs were detected with 2,000-fold diluted rabbit anti-mouse immunoglobulin HRP conjugate (RaM-HRP). The % inhibition of antigen binding due to a competing VHH or mAb was calculated as 100–100^*^ ([A450 with competing VHH or mAb] – [A450 without Ag coating]) / ([A450 without competing VHH or mAb – A450 without Ag coating]).

### Octet Red96 affinity measurements

The Octet Red96 System (Sartorius, USA) was used for affinity measurement based on Biolayer Interferometry. An assay temperature of 30 °C was used. PBS containing 0.05% Tween-20 was used as kinetics buffer in all steps of each assay. High precision streptavidin (SAX)-sensors (Sartorius) were hydrated and subsequently loaded with biotinylated M678F (2 μg/ml) for 300 s, with A/TUR 146S particles (2 μg/ml) for 900 s and kinetics buffer for 300 s (baseline step). The concentrations of FMDV particles and VHHs were optimized for affinity measurements prior to the experiments. Then association of serial dilutions of unlabeled VHHs was done for 300 s and finally dissociation for 1,800 s. A reference sensor without unlabeled VHH was included to correct for baseline drift.

The on-rate (*k*_*a*_) and off-rate (*k*_*d*_) were determined by global fitting of the association and dissociation phases of a series of unlabeled VHH concentrations. The mathematical model used assumes a 1:1 stoichiometry, fitting only one VHH in solution binding to one binding site on the surface. The equilibrium dissociation constant (*K*_*D*_), a measure for affinity, was then calculated as the ratio of *k*_*d*_ and *k*_*a*_. The Octet Analysis Studio v12.2 software (Sartorius) was used for data analysis.

### XL-MS analysis

XL-MS relies on cross-linking of protein complexes with a homobifunctional disuccinimidyl suberate (DSS) linker followed by digestion of the cross-linked complex with 5 proteases and identification of cross-linked peptides by MS analysis (30). The use of an equimolar mixture of deuterated and hydrogenated DSS cross-linker facilitates identification by MS. By tandem MS-MS the specific amino acid residues that are cross-linked can be identified. The NHS groups on the DSS bifunctional reagent only react with positively charged amino groups or OH groups, which are present on the protein N-terminus and side chains of amino acids Lys, Arg, His, Tyr, Thr and Ser (31).

XL-MS analysis was done at Coval X (Zurich, Switzerland) using SDG purified A24Cru 146S particles complexed with either M691F or M702F VHH. Strain A24Cru was used for this purpose because it produces a high amount of 75S particles in addition to 146S particles and thus could also be used for analyzing VHH binding to 75S particles. Prior to XL-MS analysis, the A24Cru 146S particles were subjected to digestion with five proteases and MS analysis (see below) without complexing with VHH and cross-linking to determine whether sufficient peptides can be identified that cover the complete FMDV capsid sequence. Such peptide mass fingerprints were made for VP1, VP2, and VP3 but not for VP4, which is internal in the FMDV 3D structure and thus not accessible to antibodies. For both VHHs 10 μl 0.5 mg/ml 146S particles were mixed with 10 μl 0.5 mg/ml VHH in PBS and 2 μl N,N-dimethylformamide containing 2 mg/ml disuccinimidyl suberate (DSS) cross-linker. The DSS consisted of an equimolar mixture of two forms that contain either 12 hydrogen (H12) or 12 deuterium (D12) atoms but were otherwise chemically identical. After incubation for 3 h at room temperature (RT) cross-linking was quenched with 20 mM ammonium bicarbonate and proteins were reduced with 50 mM dithiothreitol for 1 h at 37°C and subsequently alkylated with 100 mM iodoacetamide for 1 h at RT. Then five different aliquots of the reduced/alkylated sample were separately incubated with five different proteases (all Promega, Madison, WI, USA), overnight at 37°C (trypsin, elastase and ASP-N), 25°C (chymotrypsin) or 70°C (thermolysin). Liquid chromatography coupled to tandem mass spectrometry (LC-MS/MS) analysis was then performed. Peptides were separated on a C18 PepMapRSLC column at a flow rate of 300 nl/min ramping a gradient from 2 to 40% mobile phase B (water/acetonitrile/formic acid, 20:80:0.1) using an Ultimate 3000-RSLC system. Peptides in the range of *m/z* 350–1,700 were analyzed by LC-MS/MS using a Thermo LTQ Orbitrap XL mass spectrometer (ThermoFisher Scientific, Rockford, IL, USA). MS data were analyzed using xQuest V2.0 (32) and Stavrox V3.6 software (33) using a database of the VHH sequences and a sequence for A24Cru.

### 3D structure analysis

The A24Cru strain was sequenced again using earlier described methods (8). The protein sequence of the P1 region was identical to an earlier obtained sequence (accession number AY593768), except for VP2 residue 82 which was lysine instead of glutamic acid and VP2 residue 86 which was asparagine instead of aspartic acid (result not shown). The FMDV residues cross-linked to a VHH were mapped on the 3D structure of FMDV A22/IRQ/24/64 (A22IRQ; PDB: 4GH4) using PyMOL 2.5.2 (Schrodinger, Portland, USA). The protein sequence of A22IRQ has 90% amino acid sequence identity to A24Cru. To define an epitope, amino acid residues were selected that were closest to each other on the 3D structure.

## Results

### Epitope binning of VHHs

**Figure 1:**
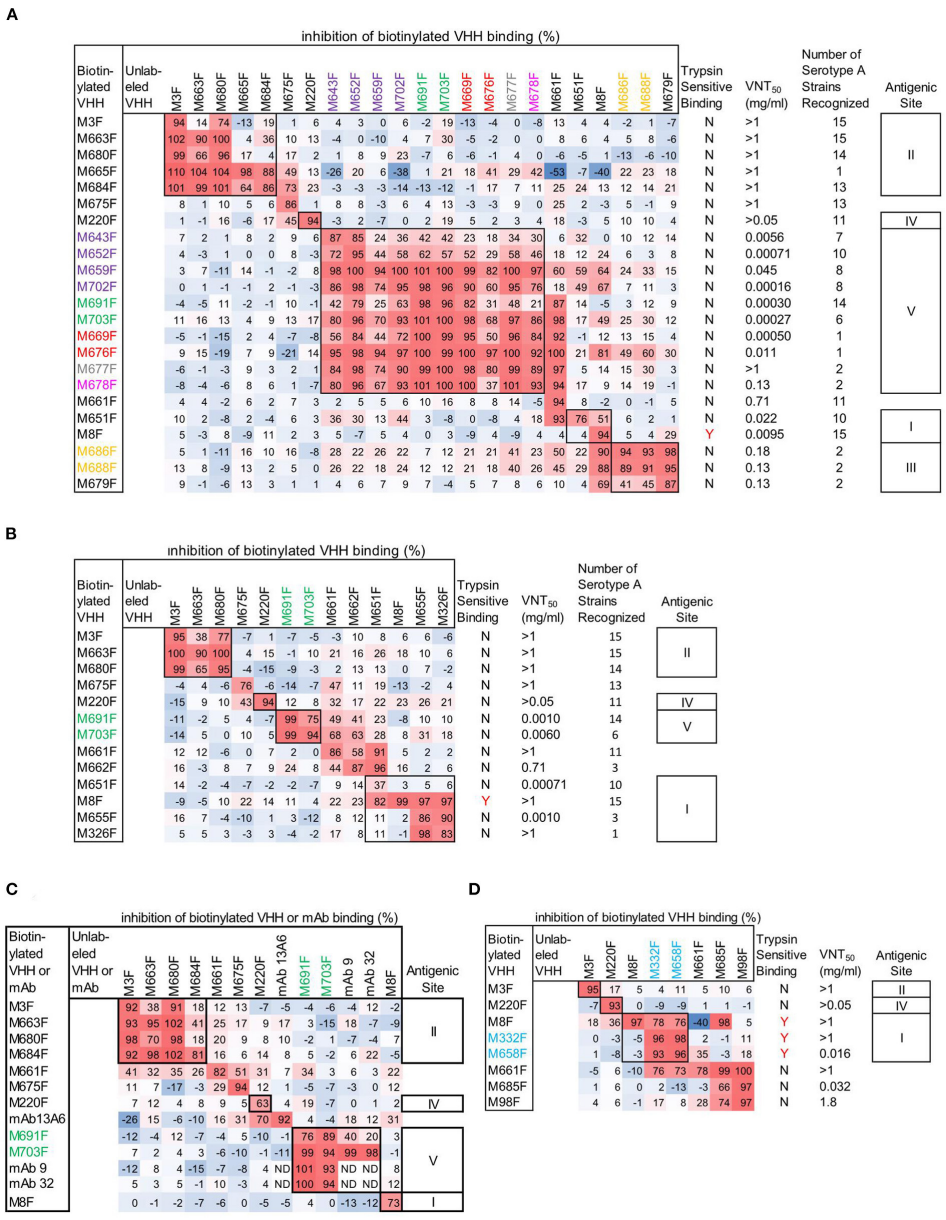
Epitope mapping of FMDV binding VHHs and mAbs by competition ELISAs using FMDV strains A/TUR **(A)**, A24Cru **(B)**, A10/HOL/1/42 **(C)**, and Asia1 Shamir **(D)**. A red-blue coloring is used to visualize differences in percentage inhibition of biotinylated VHH binding. The 14 VHHs of the 7 CDR3 groups comprising the 12 VHHs that bind specifically to 146S of serotype A or Asia1 strains are color-coded by their CDR3 group. VNT titres and binding to trypsin sensitive epitopes of VHHs is also indicated **(A, B, D)**. The number of serotype A strains recognized in ELISA by serotype A binding VHHs **(A, B)** was derived from ELISA data obtained earlier (8) using 15 serotype A strains assuming an absorbance > 0.5 indicative of binding. VHH binding is either sensitive to trypsin treatment of FMDV (Y) or not (N). Competition of mAb 9 and mAb 32 by unlabeled mAbs **(C)** could not be determined (ND) due to using RaM-HRP for mAb detection. However, mAb 13A6 was used in biotinylated form, enabling detection of competition with unlabeled mAb. Roman numerals indicate VHH epitope bins.

We mapped the antigenic sites of 30 VHHs binding to serotype A or Asia 1 FMDV by competition of biotinylated VHH with unlabeled VHH in DAS-ELISA. For competition ELISA we used FMDV strains A/TUR, A24Cru, A10/HOL/1/42 and Asia1 Shamir (Figures 1A–D). We earlier determined (5, 7, 8) the specificity of the VHHs for these strains as well as the particle specificity, which revealed 12 VHHs to be 146S specific (Table 1). Color-coding was used to indicate the seven complementarity-determining region (CDR)3 groups that contain the 12 VHHs that bind specifically to 146S of serotype A or Asia1 strains, as done earlier (8). CDR3 is the most variable region of immunoglobulin domains and most important for determining antigen binding specificity. VHHs of the same CDR3 group are most likely derived from the same B-cell but have diverged due to somatic hypermutation. VHHs M643F and M652F were not strictly 146S specific, although M652F showed high 146S specificity for C1/Detmold/FRG/60. They belong to the same (purple) CDR3 group as 146S specific VHHs M659F and M702F. VHHs M3F, M8F, and M220F were earlier found to recognize three independent antigenic sites of serotype O strain O1/Manisa/TUR/69 (26) that were indicated by roman numerals II, I, and IV, respectively (Table 1). Since these 3 VHHs cross react to serotype A and Asia1 strains, they were included in the current epitope mapping. M3F was earlier shown to be highly 12S-specific (5). We further included mAb 13A6 in this analysis since it binds to a known epitope at the VP2 N-terminus and was also found to be 12S-specific (see below).

Epitope binning of strain A/TUR (Figure 1A) was done using all 23 out of the 30 VHHs that recognize this strain. A subset of these VHHs was included in epitope binning using strain A24Cru (Figure 1B), together with A24Cru specific VHHs M326F, M655F and M662F. Furthermore, a subset of these VHHs was used in epitope binning using strain A10/HOL/1/42 (Figure 1C) that was also recognized by 146S-specific mAb 9 and mAb 32. VHHs M3F, M8F and M220F recognize three independent antigenic sites (II, I, and IV, respectively) on the three serotype A strains (Figures 1A–C), as observed earlier for type O strain O1/Manisa/TUR/69 (26). Site II was recognized by 4 further VHHs, although competition was sometimes non-reciprocal (Figures 1A–C). M675F showed non-reciprocal competition with M220F (site IV) when using A/TUR and A24Cru (Figures 1A, B) but not when using A10/HOL/1/42 (Figure 1C). VHHs M651F, M655F and M326F were mapped to site I since they efficiently inhibited biotinylated M8F binding to A24Cru, although competition was non-reciprocal (Figure 1B). However, the 146S-specific VHHs M686F and M688F, as well as 12S binding M679F were unable to compete with biotinylated M8F using A/TUR, although M8F could compete with the biotinylated versions of these VHHs (Figure 1A). We therefore mapped these 3 VHHs to an independent site (III). The remaining 8 146S-specific VHHs and M643F and M652F from the purple CDR3 group all mapped to the separate site V (Figure 1A). The non-146S specific VHHs M643F and M652F were less efficiently competed by the eight highly 146S-specific VHHs. M661F showed strong competition with many VHHs of site I and site V when used in unlabeled form but was not competed at all by any VHH when used as biotinylated VHH. Therefore, it was not grouped into an epitope bin. M8F of site I showed similar non-reciprocal competition of several site V VHHs. Notably, M661F and M8F showed 53% and 40% negative inhibition, respectively, of biotinylated M665F binding (Figure 1A). Furthermore, the two 146S-specific VHHs of site III, M686F and M688F, also showed competition with many VHHs of site V, although the percentage inhibition was lower. The 146S-specific VHHs, M691F and M703F, that bind A10/HOL/1/42 were found to compete with the earlier isolated 146S-specific mAb 9 as well as mAb 32 that was later found to bind the same antigenic site as mAb 9 (Figure 1C). MAb 13A6 showed nonreciprocal competition with M220F (site IV) only but did not compete with VHHs mapped to site II (Figure 1C) despite its similar FMDV strain and particle recognition (see below).

VHHs M3F, M8F, and M220F also recognize three independent antigenic sites (II, I and IV, respectively) on strain Asia1 Shamir (Figure 1D). The 146S-specific VHHs M332F and M658F showed non-reciprocal competition with M8F and were thus grouped to site I. The remaining Asia1 Shamir binding VHHs were difficult to map. M661F again showed non-reciprocal competition with many VHHs and 40% negative inhibition of M8F binding.

### Virus neutralization and FMDV strain recognition of VHHs

Epitopes were further characterized by VNT titers (Figures 1A, B, D) and broadness of serotype A strain (*n* = 15) recognition (Figures 1A, B). VHHs that recognized sites II and IV as well as M675F that sometimes competed with M220F of site IV consistently did not neutralize FMDV while they displayed broad recognition of serotype A strains (11 to 15) with exception of M665F that bound only one serotype A strain. Nine out of 10 VHHs recognizing site V were neutralizing. Neutralizing titers were generally lower using strain A/TUR than A24Cru and also lower using VHHs that recognized more serotype A strains. Among the eight Asia1 Shamir binding VHHs, only three VHHs showed virus neutralization, including M658F which recognized antigenic site I and 2 VHHs that were difficult to map (Figure 1D).

### Binding to trypsin sensitive epitopes and VP2 peptide recognition of VHHs

To further characterize the epitopes, we also determined the binding to trypsin sensitive epitopes for all 30 VHHs by incubation of dilution series of untreated and trypsin-treated antigen (TTA) in ELISA (Supplementary Figure 1). VHHs were considered binding to trypsin sensitive epitopes (Figures 1A, B, D) if the FMDV antigen concentration resulting in an absorbance value of 0.2 in dilution series of antigens (EC_0.2_) was at least tenfold higher in trypsin-treated samples as compared to untreated samples. Using this criterium only VHHs M8F, M332F, and M658F showed trypsin sensitive binding (Figures 1A, B, D). This is consistent with the classification of M332F and M658F to antigenic site I for Asia1 Shamir but not consistent with the classification of M651F, M655F, and M326F to antigenic site I for A24Cru.

Since only the Asia1 Shamir 146S-specific VHHs M332F and M658F fell into the same epitope bin as M8F, we titrated both untreated FMDV antigen consisting mostly of 146S and heated antigen (12S) in the M8F ELISA. M8F showed considerable 146S specificity for strain Asia1 Shamir (Supplementary Figure 2C) although not as high as M332F (Supplementary Figure 2M). Consistent with earlier results (8) 146S specificity for serotype A or O strains was much lower (Supplementary Figures 2A, B, D). MAb 13A6 showed a high 12S specificity for 4 FMDV strains from serotypes A, O and Asia 1 that was comparable to M3F (Supplementary Figures 2E–L).

None of the VHHs bound in ELISA to a peptide representing the N-terminus of VP2 conjugated to BSA (Supplementary Figure 3A) although they did bind at least one of the FMDV strains used also for epitope binning (Supplementary Figures 3C–E). However, mAb 13A6 was able to bind this peptide as it reacted well with VP2-22-BSA but not with BSA (Supplementary Figures 3A, B).

The above classification of antigenic sites is summarized in Table 2.

**Table 2:** Characteristics of antigenic sites recognized by VHHs.

**Anti-genic site^a^**	**FMDV serotype specificity^b^**	**Prototype VHHs for different FMDV serotypes**			**Antigenic site identified using mAbs**	**FMDV particle specificity^c^**	**Neutra-lizing^e^**	**Trypsin sensitive epitope^f^**
		**O**	**A**	**Asia1**				
I	Low (M8)	M8F	M8F	M8F, M332F	1	(12S and) 146S^d^	Yes	Yes
II	Low	M3F	M3F	M3F	unknown	12S	No	No
III	High	M23F	M686F, M688F	-	unknown	(12S and) 146S	Yes	No
IV	Low	M220F	M220F	M220F	unknown	12S and 146S	No	No
V	High	-	M691F, M702F	-	2 and/or 4	146S	Yes	No

^a^Sites I to IV were initially identified in serotype O FMDV (26); site V, this work.

^b^Low, reactivity with at least 2 serotypes; High, reactivity with strains from 1 serotype only.

^c^See Table 1.

^d^M8F and M332F specifically bind 146S particles of Asia 1 Shamir, whereas M8F recognizes 12S of serotype O and A strains.

^e^Neutralizing at least one of the strains tested.

^f^At least one of the VHHs mapped to this site binds to a trypsin sensitive part of the epitope.

### Affinity of VHHs M691F and M702F

**Figure 2:**
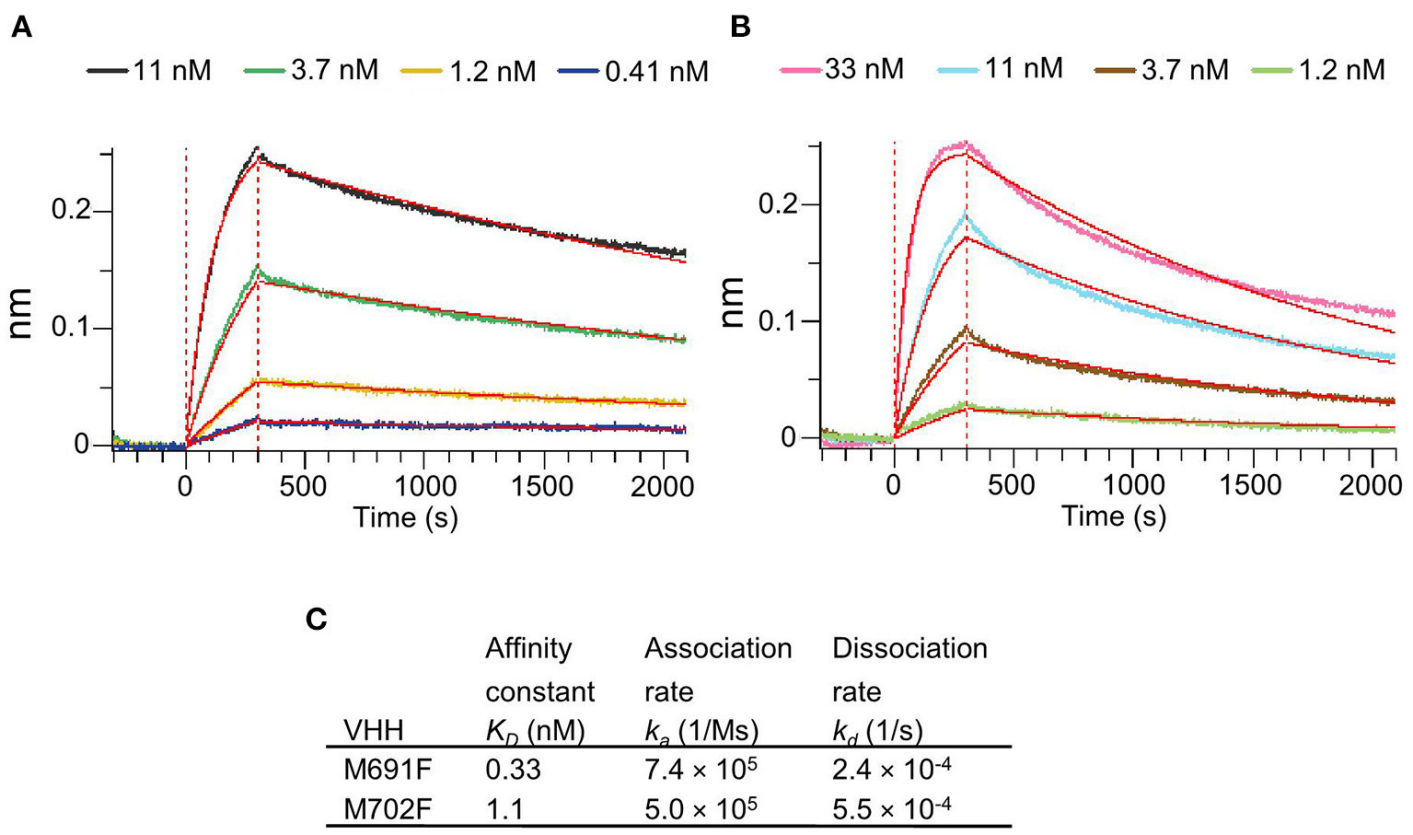
Affinity binding curves and deduced affinity constants of VHHs. Using Biolayer Interferometry on an Octet Red96 system, association and dissociation rates were determined by tight multivalent capturing of FMDV A/TUR on optical streptavidin sensors that were loaded with biotinylated M678F VHH. The FMDV-bound sensors were incubated with specific concentrations of M691F **(A)** or M702F **(B)** to allow association at time = 0 s. After 300 s the sensors were then moved to VHH-free solution and allowed to dissociate over a time interval. Curve fitting using a 1:1 interaction model (red lines) allows for the affinity constant (*K*_*D*_) to be measured for each VHH **(C)**.

The VHHs M691F and M702F that bound site V were selected for further study because of their high specificity for intact particles and broad strain recognition. Their affinity for FMDV strain A/TUR was determined by biolayer interferometry using an Octet Red96 biosensor. Both VHHs bound 146S particles with high affinity as shown by the low *K*_*D*_ values of 0.33 nM for M691F and 1.1 nM for M702F (Figure 2).

### Epitope mapping by XL-MS

We started the XL-MS analysis using A24Cru 146S particles without complexation to VHH. All three VPs had a high sequence coverage in the peptide mass fingerprints (Supplementary Figure 4) that varied from 96.7 to 98.6% (Supplementary Table 1). Only the DSS reactive residues VP1-K210, VP3-S203, and VP3-S205 were not represented in the peptide mass fingerprints (Supplementary Figure 4).

**Figure 3:**
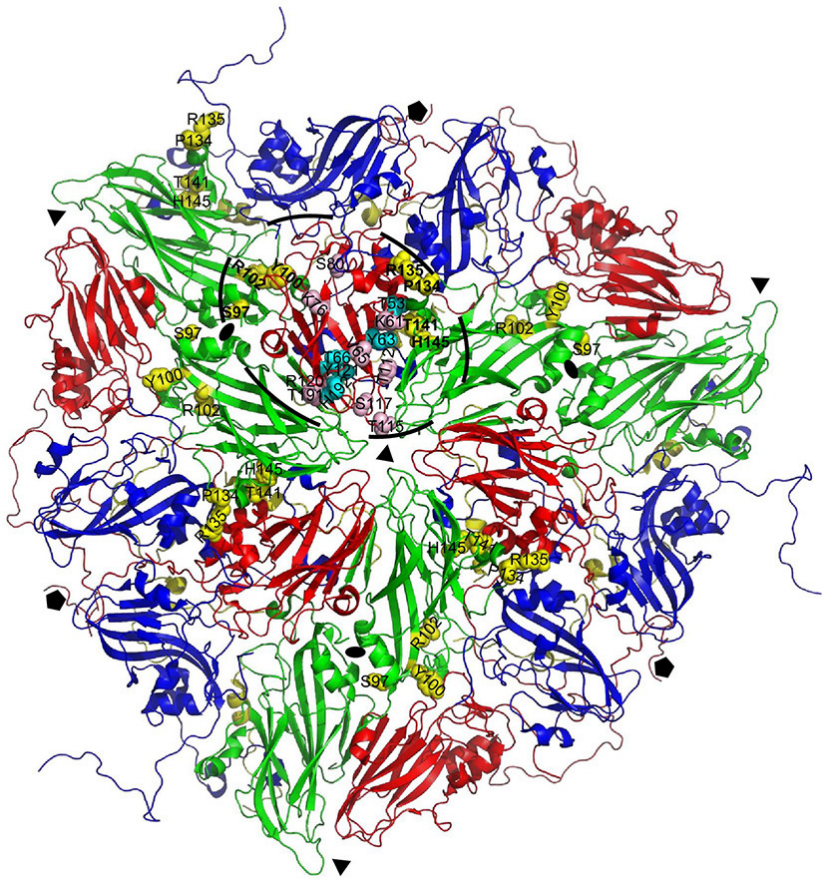
Location of VP2 residues cross-linked to M702F relative to VP3 cross-linked residues. Cross-linked residues are mapped onto a cartoon presentation of the A22IRQ structure (PDB: 4GH4). Six protomers that originate from three different 12S pentamers surrounding the 3-fold symmetry axis are shown. M702F cross-linked residues are shown for VP3 of the protomer on top and the 4 VP2 molecules closest to this VP3 molecule. The VP2 residues closest to the cross-linked VP3 residues (bold) most likely form the same antigenic site (dashed oval). VP1, blue; VP2, green; VP3, red; VP4, yellow. Cross-linked residues are shown with side chains as yellow (VP2) or cyan/pink (VP3) spheres. Symmetry axes: 5-fold, pentagon; 3-fold, triangle; 2-fold, oval.

XL-MS analysis of M691F and M702F complexed to 146S particles identified several A24Cru amino acid residues cross-linked to a VHH (Table 3). M702F was cross-linked to residues in VP2 and VP3 whereas M691F was cross-linked to residues in VP1, VP2, and VP3. We mapped the cross-linked residues onto the 3D structure of the A22IRQ 146S particle. Residues VP1-141 and 149, VP2-134 and VP3-65 are different in A22IRQ compared to A24Cru (Table 3). We further refer to these residues based on the corresponding residues in A22IRQ. The residues cross-linked to M702F were visualized in cyan or pink for a single VP3 molecule on the A22IRQ 3D structure showing six protomers around the 3-fold axis (Figure 3). Most cross-linked residues were located close to the 3-fold symmetry axis where three 12S pentamers associate. All seven residues cross-linked to VP2 were marked in yellow on 4 of the 6 VP2 molecules surrounding the VP3 molecule with marked cross-links (Figure 3). Residues P134, R135, T141, and H145 located on the VP2 molecule of an adjacent protomer shown at the right of the VP3 molecule with visualized 16 cross-linked residues were clearly closest to these VP3 residues. However, S97, Y100, and R102 shown on the right on this same VP2 molecule were located far away from these marked VP3 residues, whereas these same 3 residues of the VP2 molecule of the same (top left) protomer were closest to these VP3 residues and thus most likely part of the same M702F antigenic site.

**Table 3:** Cross-linked amino acid residues (X) of FMDV A24Cru 146S particles complexed with M702F or M691F VHHs identified by XL-MS analysis.

**Cross-Linked residue A24Cru**	**Corresponding Amino acid Residue**		**146S Particle surface exposed^b^**	**Amino acid residues of VHHs cross-linked (IMGT position)** ^**c**^																			
				**M702F**												**M691F**							
				**CDR1**				**FR2**	**CDR2**		**CDR3**					**CDR1**		**FR2**	**CDR2**	**CDR3**			
				**(27–38)**				**(39–55)**	**(56–65)**		**(105–117)**					**(27–38)**		**(39–55)**	**(56–65)**	**(105–117)**			
	**A22IRQ**	**O1K^a^**		**S26**	**S29**	**S31**	**Y33**	**R55**	**S59**	**T62**	**S109**	**T111**	**S113**	**R114**	**Y117**	**S26**	**T34**	**T55**	**S61**	**K105**	**T111**	**T116**	**Y117**
***VP1***																							
S141	T141	V141	Yes													X	X				X		X
R143		L144	Yes																			X	
S149	P149	V150	Yes																			X	
***VP2***																							
S97			Yes															X					
Y100			Yes												X						X		
R102			Internal					X	X						X		X			X			
T134	P134	K134	Yes				X							X	X								
R135			Yes												X								
T141			Internal	X							X												
H145			Internal								X										
H209			Inner surface																	X		X	
***VP3***																							
T53			Internal						X	X													
K61		V61	Yes						X	X													
Y63			Yes						X														
T65	V65		Yes							X													
T66			Yes						X														
T68			Yes																	X	X		
R72			Yes																			X	
K76		Q76	Yes						X													
S80			Internal	X																			
H85			Yes													X							
T112			Yes										X										
T115			2-fold axis						X														
S117		A117	Internal						X						X								
R120			2-fold axis						X														
Y121			Internal							X												
Y170		(Y169)	Yes																X				
T191		(T190)	2-fold axis	X	X	X		X														
H192		(H191)	Yes			X	X																
T199		A198	Yes													X						
K208		(K207)	Inner surface																		X		

^a^Due to an insertion at VP3 position 142 of O1K as compared to serotype A strains residues between parentheses are identical in O1K and A24Cru. RGD is position 145-147 in O1K and 144-146 in A22IRQ.

^b^Residues are classified as exposed at the outer surface of the 146S particle (yes), exposed at the inner surface, exposed at the 2-fold axis of a 12S pentamer only, or internal.

^c^The IMGT system (34) was used to define the three CDRs often involved in antigen binding, the second framework region (FR2) of VHHs, and VHH numbering.

Some VHH residues were complexed to different FMDV residues. This was especially the case for M702F residue S59, that was cross-linked to 9 different FMDV residues, but also S26, T62, and Y117. The contact residues for all these four M702F residues are spread over a large area covering both VP2 and VP3 that exceeds twice the 11.4 Å length of the DSS spacer arm (Supplementary Figure 5).

**Figure 4:**
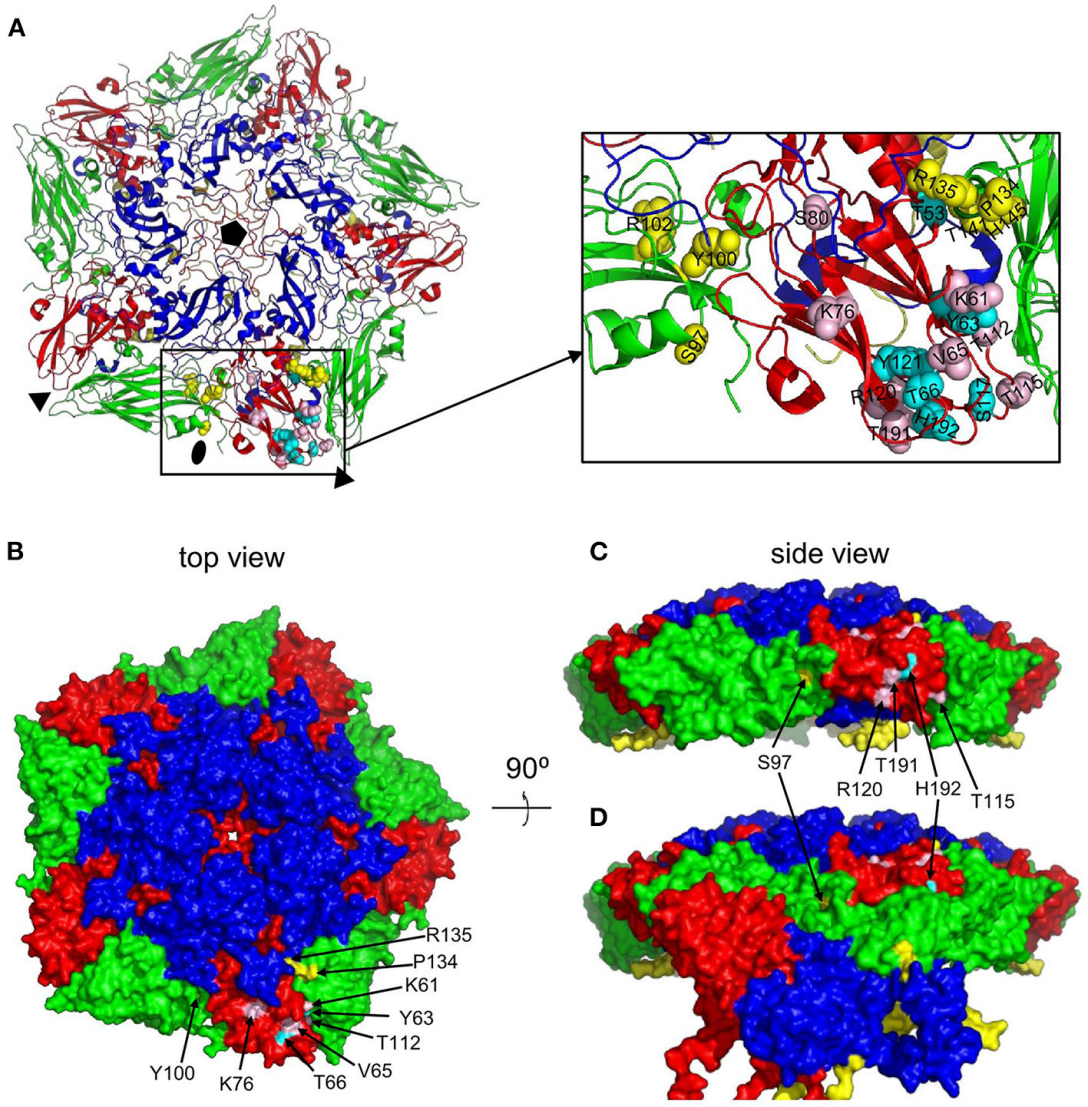
Surface accessibility of M702F cross-linked residues mapped on a 12S pentamer of the A22IRQ structure (PDB: 4GH4). The M702F cross-linked residues forming a single antigenic site on VP3 of the protomer at the bottom and the relevant VP2 residues adjacent to this VP3 molecule are shown as cartoon graph **(A)**. Cross-linked residues are shown with side chains as yellow (VP2) or cyan/pink (VP3) spheres. The surface accessible area of a pentamer is shown from a top view **(B)** or side view **(C, D)**. The nine residues that are surface accessible even when 12S pentamers associate are indicated by arrows **(B)**. Cross-linked residues surface exposed at the 2-fold symmetry axis are shown by a side view of a single pentamer **(C)** or this same pentamer with a protomer of an adjacent 12S pentamer that associates at the 2-fold symmetry axis in a 146S particle **(D)**. Among the 5 cross-linked residues at this 2-fold symmetry axis (arrows), R120, T191 and T115 are only surface accessible in a 12S pentamer **(C)** but not a 146S particles **(D)**. VP1, blue; VP2, green; VP3, red; VP4, yellow. Fivefold symmetry axis, pentagon; 3-fold symmetry axis, triangle; 2-fold symmetry axis, oval.

The putative M702F antigenic site was located on a single 12S pentamer, close to the 2-fold symmetry axis that separates two pentamers (Figure 4A). VP3-K61, Y63, V65, T66, K76, T112, and VP2-Y100, P134, R135 are surface exposed when looking at a pentamer from a top view (Figure 4B), although in case of VP2-Y100 only the OH group is exposed. VP3-T115, R120, T191, H192, and VP2-S97 are surface exposed when looking at a pentamer from a side view toward the 2-fold symmetry axis (Figure 4C). However, VP3-T115, R120, and T191 are not surface exposed in a 146S particle when this 2-fold symmetry axis is blocked by a protomer of an adjacent 12S pentamer, whereas VP3-H192 and VP2-S97 are also surface accessible in 146S particles (Figure 4D). Surprisingly, cross-linked residues VP2-R102, T141, H145 and VP3-T53, S80, S117, Y121 are not surface accessible but internal in a 12S pentamer. However, these residues are mostly located close to surface accessible cross-linked residues (Figure 4A): VP2-R102 is close to VP2-Y100; VP2-T141, VP2-H145, and VP3-T53 are located close to VP2-P134 and R135. Similarly, VP3-S117 and Y121 are located close to surface exposed VP3-V65, T66, and H192. VP3-S80 is buried under the C-terminus of VP1. The surface accessibility of cross-linked residues is summarized in Table 3.

**Figure 5:**
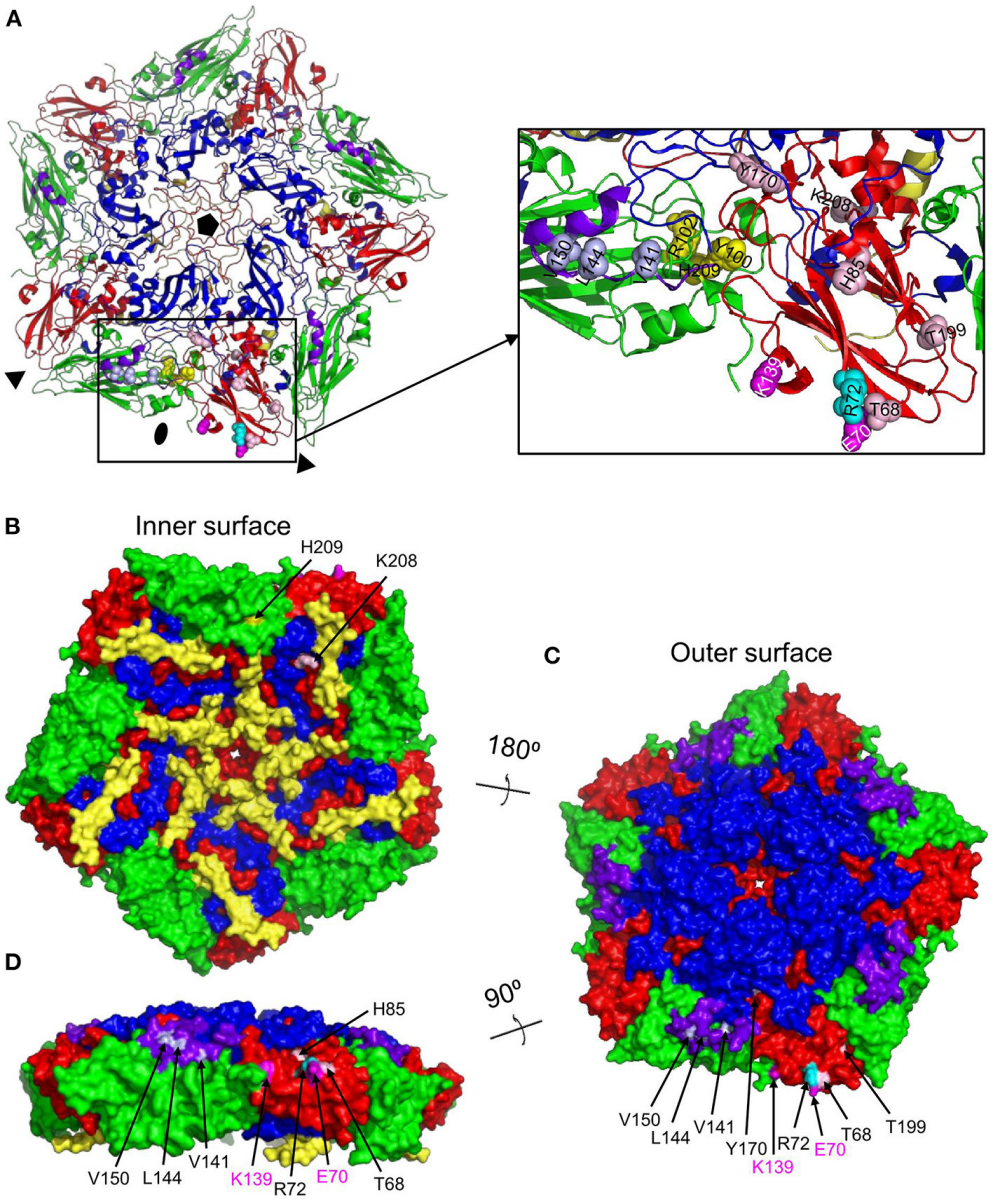
Surface accessibility of M691F cross-linked residues mapped on a 12S pentamer of the A22IRQ structure (PDB: 4GH4) with superimposed GH loops of reduced O1K (PDB: 1FOD). The M691F cross-linked residues forming a single antigenic site on the protomer at the bottom is shown as a cartoon graph **(A)**. Side chains of cross-linked residues are shown as light blue (VP1) yellow (VP2) or cyan/pink (VP3) spheres. The surface accessible area of a pentamer **(B, C)** is shown for the inner surface **(B)**, outer surface **(C)** or from a side view looking at the 2-fold symmetry axis **(D)**. The 8 residues that are surface accessible are indicated by arrows **(B–D)**. VP1, blue; VP1 GH-loop, purple; VP2, green; VP3, red; VP4, yellow. The side chains of VP3-K139 and VP3-E70 that are mutated in mAb 9 escape mutants are indicated in magenta spheres. Fivefold symmetry axis, pentagon; 3-fold symmetry axis, triangle; 2-fold symmetry axis, oval.

The M691F antigenic site was also mapped onto the A22IRQ 3D structure. However, since cross links were observed with the highly flexible VP1 GH-loop, which is disorded and thus invisible in the A22IRQ structure, we introduced the O1 Kaufbeuren (O1K) GH-loop that could be resolved by X-ray crystallography due to reducing the disulfide bond involving C134 at the base of the GH loop (35). The putative M691F antigenic site was also located on a single protomer of a 12S pentamer (Figure 5A), on a similar position as the M702F antigenic site (cf. Figure 4A).

Cross-linked residues VP2-H209 and VP3-K208 are surface accessible only at the inner surface of 12S pentamer (Figure 5B) which is not accessible in a full 146S particle. VP1-V141, L144, V150, and VP3-T68, R72, H85, Y170, and T199 are surface exposed in a 146S particle, without being blocked by an adjacent pentamer (Figures 5C, D). VP2-Y100 was also cross-linked to M702F and surface accessible without artificial introduction of the O1K GH-loop (Figure 4B) but is hidden by the reduced O1K GH-loop (Figure 5C) which is known to lie flat on the virion surface, as opposed the natural GH loop that stands up from the surface (35). VP2-R102 was also cross-linked to M702F. It is internal, but close to surface accessible residue Y100. Although VP2-H209 is only accessible from the 12S pentamer inner surface (Figure 5B), its localization close to VP2-R102 and Y100, which were cross-linked to both M691F and M702F, suggests that its cross-linking is not an artifact. The surface accessibility of residues cross-linked to M691F is summarized in Table 3.

Residues VP3-E70 and K139 that were mutated in mAb 9 resistant mutants (21) are also located close to the 2-fold symmetry axis (Figure 5). E70, which is most important for mAb 9 binding, is close to M691F cross-linked residues VP3-T68 and R72, which are part of the VP3 BC-loop.

### Comparison of epitopes identified by XL-MS to M170 epitope identified by cryo-EM

**Figure 6:**
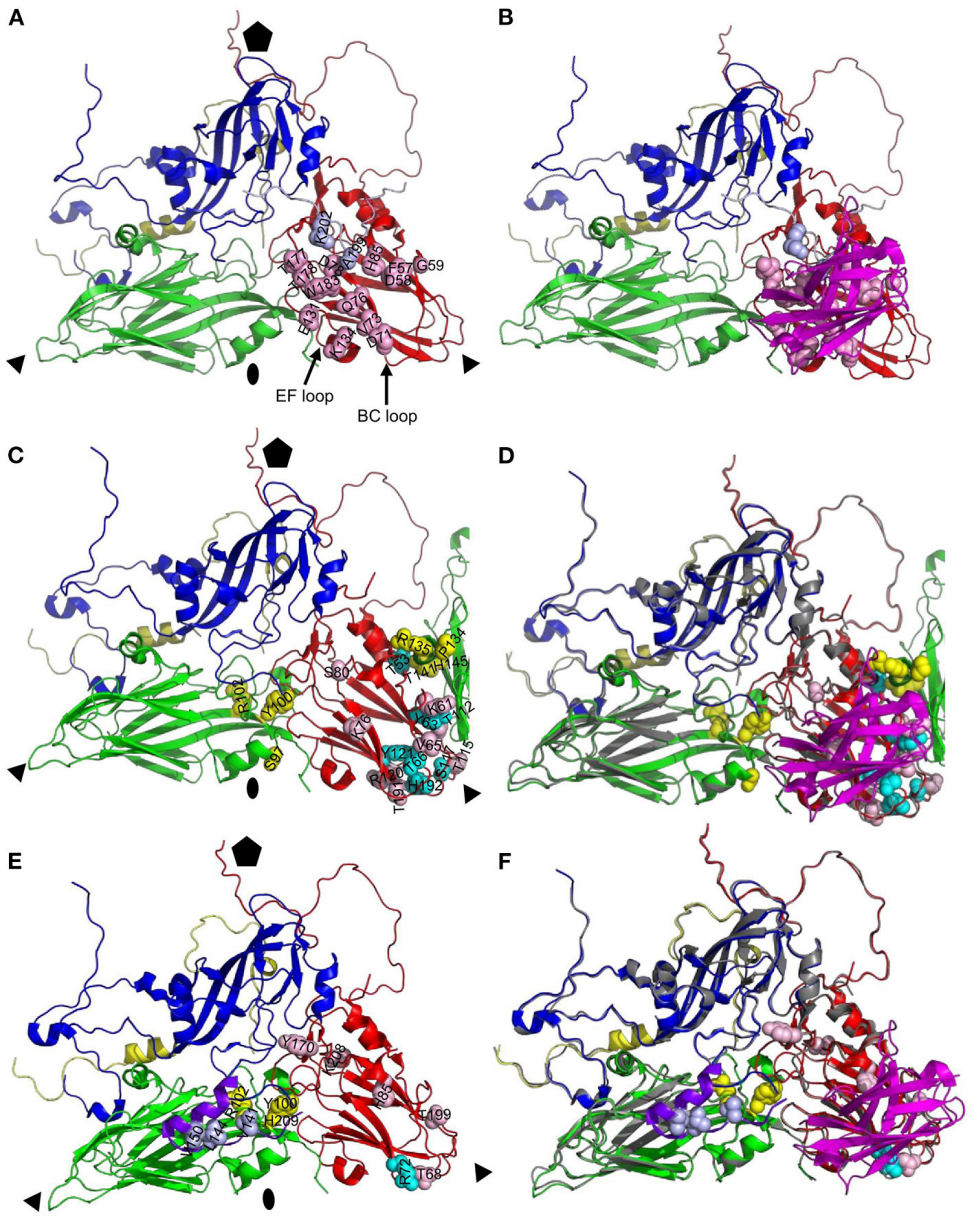
Comparison of antigenic site of VHH M170 determined by cryo-EM and M702F and M691F determined by XL-MS. Protomers of O/BY/CHA/2010 [PDB: 7DST; **(A, B)**] or A22IRQ [PDB: 4GH4; **(C–F)**] are shown as cartoon with footprints of M170 **(A, B)**, M702F **(C, D)** or M691F **(E, F)** shown by indicating the side chains of residues within 4 Å of the VHH **(A, B)** or cross-linked to the VHH **(C–F)** as light blue (VP1), yellow (VP2), or cyan/pink (VP3) spheres. VPs and VHHs are shown as cartoon: VP1, blue; VP1 of O1K GH-loop **(E, F)**, purple; VP2, green; VP3, red; VP4, yellow; M170, magenta. The C-terminal 20 amino acids of VP1 from an adjacent protomer are shown as light blue cartoon to visualize the M170 footprint **(A, B)**. Part of VP2 from an adjacent protomer is shown to visualize the M702F cross-linked residues **(C, D)**. An alignment of the structures of A22IRQ (VP coloring as done throughout this paper) with M702F or M691F cross-linked residues and O/BY/CHA/2010 (gray) complexed to M170 is shown **(D, F)**. Fivefold symmetry axis, pentagon; 3-fold symmetry axis, triangle; 2-fold symmetry axis, oval.

The epitope recognized by M170 on type O FMDV as resolved by cryo-EM of M170/FMDV complex (10) was visualized on a single protomer, including the C-terminal 20 amino acids of VP1 from an adjacent protomer, since M170 interacts with two residues in this region (Figures 6A, B). For comparison the epitopes recognized by M702F and M691F as identified by XL-MS were visualized in an identical manner on an A22IRQ protomer, including part of VP2 of an adjacent protomer in case of M702F, since cross-links presumably occur to this region (Figures 6C, E). The M170 footprint is located mostly on VP3 and includes two residues on the VP1 C-terminus of an adjacent protomer. Superposition of the M170/FMDV cryo-EM structure and the A22IRQ structure visualizing the cross-links to M702F or M691F reveals M170 in the middle of the residues cross-linked to M702F or M691F (Figures 6D, F). Note that the GH loop of O1K superimposed on this structure visualizing the M691F cross-links is in a “down” conformation and thus more distant to the other cross-linked residues as when this loop was in an “up” conformation, similar as observed when complexed to M8 (10). Taken together, this superposition confirms that the sites recognized by M170, M702F and M691F largely overlap. Several residues of the M170 epitope, such as D71 and V73 in the BC loop, and E131 and K134 in the EF loop, lie in the region with the largest conformational changes between 12S and 146S (Figure 6A). This region is likely also part of the M691F and M702F epitopes, explaining their 146S specificity.

## Discussion

The antigenic sites of type A and Asia1 FMDV binding VHHs were characterized by competition ELISA, virus neutralization tests, trypsin sensitivity of the epitope, broadness of the serotype A recognition and, for 2 VHHs, XL-MS. In competition ELISAs some VHHs showed non-reciprocal competition, which complicates antigenic site mapping. In case of M8F this could be due to VHH binding causing structural changes in the FMDV capsid, since M8 binding is known to decrease the virion stability and stimulate viral uncoating (10). Such effects can also explain the negative inhibition observed with some VHHs, especially M665F (Figure 1A), which shows improved binding to FMDV antigen complexed with a surplus of M8F. Such cooperative binding is more often observed, for example with tetanus toxin binding antibodies (36). M661F, which also shows cooperative binding of M665F and non-reciprocal inhibition of several 146S-specific VHHs (Figures 1A, B, D), possibly also causes virion structural changes. The non-reciprocal competition of several 146S-specific VHHs by M643F and M652F is consistent with the binding of 12S particles by these latter VHHs since the antigen used in the competition ELISAs was not purified by SDG and thus contained 12S in addition to 146S. Non-reciprocal competition can also be caused by differences in antibody affinity. The high affinity of M691F (*K*_*D*_= 0.33 nM) probably explains some of the non-reciprocal competition seen with M691F, which shows less than 50% inhibition by VHHs M659F, M676F, M677F, M678F, and mAb 9 while inhibiting the binding of these 5 VHHs/mAb for >98% (Figures 1A, C). Taking the above considerations into account, we mapped the type A VHHs into sites I, II, IV and V and type Asia1 VHHs into sites I, II and IV. In this mapping we also considered the broadness of FMDV strain recognition, virus neutralization and trypsin sensitivity of the epitope recognized by the VHHs (Table 2). However, it should be noted that some VHHs, such as M675F and M661F, could not be mapped into these antigenic sites due to different maps obtained with different FMDV strains.

The 12S-specific non-neutralizing VHH M3F was found to bind a different antigenic site than mAb 13A6. Furthermore, M3F did not bind to a trypsin sensitive epitope and did not bind a peptide representing the VP2 N-terminus. Freiberg et al. (24) were surprised to find that mAb 13A6 is suitable for detection of FMDV antigens in ELISA since it is specific for the VP2 N-terminus, which is hidden internally in a full capsid. We have shown in this study that mAb 13A6 shows considerable preference for 12S particles. Thus, the exposure of the VP2 N-terminus in 12S particles but not 146S particles explains the particle specific binding by mAb 13A6. Most likely the antigens used by Freiberg et al. (24) also contained some 12S. A 12S-specific mAb (23KF-1) that binds strains from all six serotypes except SAT2 was identified earlier (37). Possibly the epitope recognized by M3F is similar to the epitope recognized by mAb 23KF-1 as M3F also does not bind SAT2 strains while binding strains from serotypes O, A, C and Asia1 (7). Taken together, at least two independent 12S specific antigenic sites exist, one of them being the trypsin sensitive VP2 N-terminus while the other site is not trypsin sensitive and located in an unknown position.

**Figure 7:**
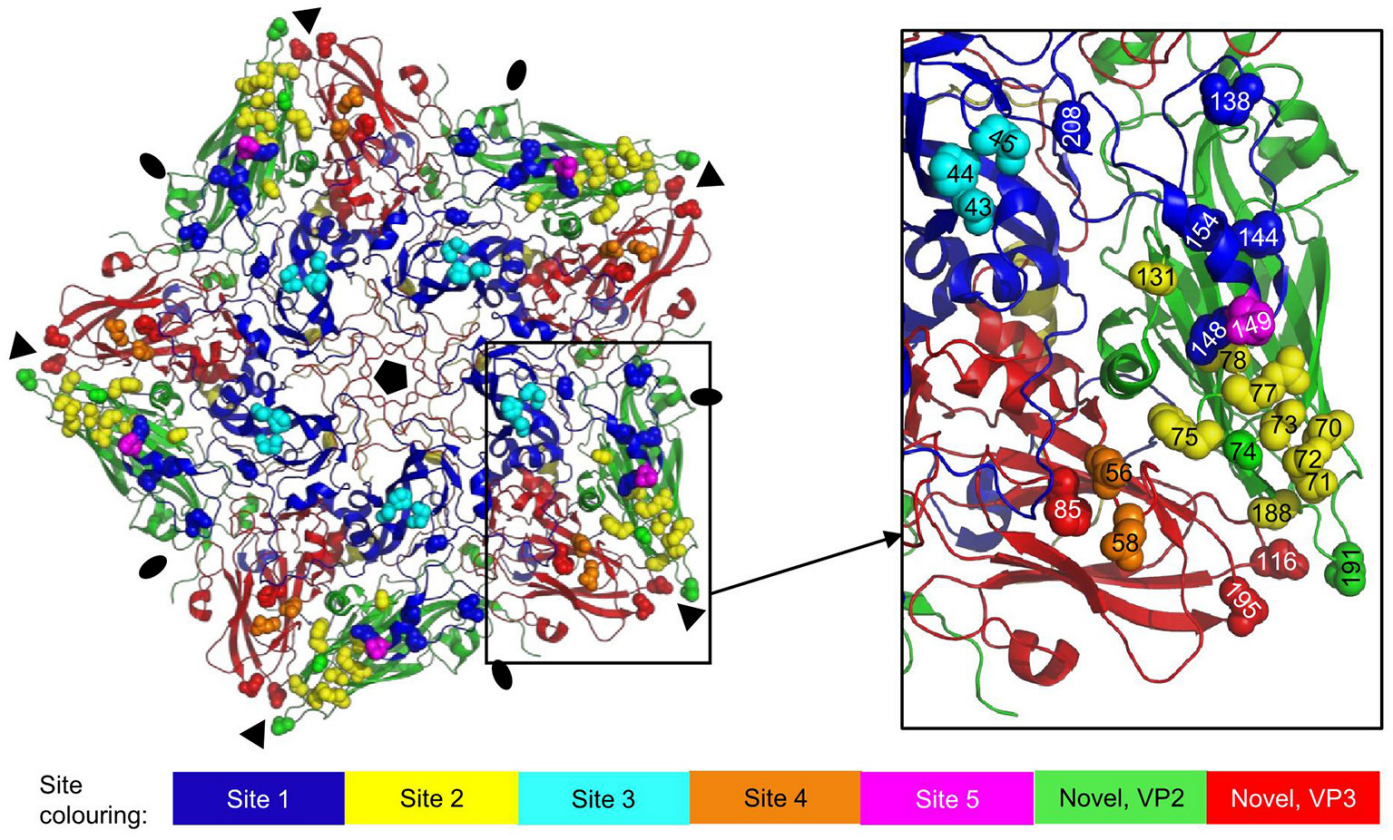
Antigenic sites 1-5 as earlier identified by sequence analysis of mAb escape mutants mapped onto the 3D structure of an O1K (PDB: 1FOD) pentamer. VPs are shown as cartoon: VP1, blue; VP2, green; VP3, red; VP4, yellow. Side chains of residues defining the antigenic sites are shown as spheres in different colors for sites 1-5 and in case of the novel site (16, 38) also VP2 and VP3 residues. Fivefold symmetry axis, pentagon; 3-fold symmetry axis, triangle; 2-fold symmetry axis, oval.

Most type A 146S-specific VHHs bound site V whereas M686F and M688F bound a separate site, that was given roman numeral III, similar to the site identified in type O by M23F (26), since M23F is highly strain specific, although we do not know whether these sites in type O and A cover a similar epitope. Thus, 146S specificity of type A binding VHHs relies on at least two separate antigenic sites. The two serotype Asia1 146S-specific VHHs, M332F and M658F, presumably recognize site I based on competition with biotinylated M8 and binding to trypsin-sensitive epitopes. Based on the previously mapped M8 epitope (10), site I encompasses predominantly the VP1 GH-loop, and is thus similar to site 1 identified in type O using conventional mAbs (12, 13) which is identical to site A in type A FMDV (9, 19). We mapped the type O antigenic sites 1-5 onto the O1K 3D structure (Figure 7). Antibodies binding site 1 generally recognize both 12S and 146S particles, which was also observed for M8 using type A and O strains (8) and is consistent with this site being a linear epitope (23, 26). However, site 1 of type Asia1 is conformational (39), explaining the 146S-specific binding of M8F to strain Asia1 Shamir that we observed here. Several 146S specific mAbs binding Asia1 Shamir were earlier reported to bind two independent antigenic sites as assessed by competition ELISAs and isolation of MAR mutants (40), that were not sequenced, and thus cannot be coupled to known antigenic sites.

The 146S-specific VHHs M691F and M702F both recognized antigenic site V. Their epitopes were further mapped by XL-MS. Several VHH residues were cross-linked to different FMDV residues that are located at a distance that far exceeds the 11.4 Å length of the spacer arm of the DSS cross-linker used. This could be due to the VHH-FMDV complexes adopting different conformations. Such flexibility was earlier reported for complexes of FMDV with VHHs M8 and M170 (10) or integrin receptors (41). FMDV structural flexibility probably also explains the cross-linking to residues that are not surface exposed in the capsid 3D structure. Structural flexibility is more often reported for other picornaviruses than FMDV. Poliovirus undergoes a global conformational change upon receptor binding, characterized by a 4% expansion. These 135S particles can adopt further different conformations (42). Cross-linked FMDV residues are not necessarily making contact with the VHH. Due to the use of a chemical cross-linker with a relatively long 11.4 Å spacer arm, residues that are quite far apart can be cross-linked (43). Such effects can at least partly explain the relative large area covered by the M691F or M702F footprints identified by cross-linked residues as compared to the M170 epitope resolved by cryo-EM (Figure 6). Furthermore, the M691F residues cross-linked to the VP1 GH-loop are probably closer to the VP3 cross-linked residues than displayed in Figures 5A, 6E since here the reduced O1K GH-loop was used, which lies flat on the virion surface. Nevertheless, the footprints of M691F and M702F clearly overlapped with the M170 epitope, and included cross-links to the VP3 BC-loop that showed a different conformation in 12S and 146S particles, explaining the 146S-specific binding of M170 (10). Most notably, we believe all cross-links observed are located on a single pentamer without crossing the 2-fold symmetry axis, on an adjacent pentamer (Figure 3), because no cross links were found to VP2 residues close to the 2-fold axis opposing the VP3 cross-linked residues of an adjacent 12S pentamer. Thus, 146S specific binding of M691F and M702F is unlikely caused by dissociation of the epitope due to dissociation of 146S- into 12S particles and more likely due to altered structures of the epitopes due to 146S particle dissociation, similar to M170. This most likely also applies to mAb 9. The bovine mAb R55 neutralizing A/WH/09 was recently described (44). The cryo-EM structure of the FMDV-R55 complex showed R55 binding to two adjacent pentamers close to the 3-fold axis. The cross pentamer binding was strongly dependent on VP3-E70 and caused virus neutralization due to prevention of virus dissociation and genome release. The binding of mAb R55 to 12S particles was not previously reported.

The site recognized by 146S-specific VHHs M170, M691F, and M702F overlaps with antigenic sites 2 and 4, which lie close together (Figure 7) and are known to interact (15). In addition to sites 1-5, a novel neutralizing site was reported for type O, encompassing VP2-74 and 191 (16) and VP3-85, 116 and 195 (38), that is conserved in type A (17). This site is also located close to sites 2 and 4, and close to the 3-fold axis (Figure 7). The 146S-specific binding of mAbs is often not thoroughly discussed in literature on FMDV antigenic sites even when such data are present (9, 22). Two mAbs against type A FMDV that recognize the same antigenic site were specific for 146S particles and had escape mutations at VP1-H201 (mAb 2PE4) or VP3-T178 (mAb 2PD11) (18). Due to insertions/deletions between types A and O, these residues correspond to VP1-202 and VP3-177 in type O, which are contact residues for M170 (Figure 6D). MAb C8 and mAb C9 against type O recognize independent antigenic sites and were specific for 146S particles (45). They were later (13) found to have escape mutations at VP2-72 (C9) and VP1-43 and 44 (C8), which corresponds to sites 2 and 3, respectively (Figure 7). MAb S11B that binds type O site 3 also appears to be 146S specific (46). Thus, for type O, also two independent antigenic sites exist that are 146S specific, as we observed also for type A. The 146S-specific binding by mAbs against site 3 is surprising since it is distant from the 2-fold symmetry axis (Figure 7).

M702F was cross-linked to VP3 residues T115, R120 and T191 at the 2-fold symmetry axis surface that is accessible in a 12S particle, but not in a 146S particle. Holes in the viral capsid have earlier been observed at the 2-fold axis of other picornaviruses, including poliovirus 135S particles (42) and acidified Seneca Valley virus, which resulted in a major reconfiguration of the pentameric capsid assemblies, resembling a potential uncoated intermediate (47). The cross-linking of an 146S-specific VHH to residues that are hidden at the 2-fold symmetry axis of rigid 3D models of 146S particles is probably again explained by flexibility of virions in solution, with holes at the 2-fold axis. Such cross-linking to residues buried at the interface of two 12S particles is not observed for M691F. Possibly, the difference in recognition of full and empty particles by M691F and M702F (8) relies on structural differences between these particles at the 2-fold symmetry axis.

## Conclusions

We have shown for type A FMDV that both 12S and 146S particle specificity relies on at least two independent antigenic sites. The major 146S specific antigenic site that is also recognized by the VHHs M691F and M702F was located close to the 2-fold and 3-fold symmetry axes on a similar position as the type O 146S specific VHH M170. Since this site was located on a single 12S particle the 146S-specific binding of M691F and M702F is probably also caused by different conformations of 12S and 146S particles, as earlier suggested for M170 (10). The cross-linking of FMDV residues that are not surface exposed in 146S particles (M691F and M702F), or only surface exposed in 12S particles but not in 146S particles (M702F) suggests that FMDV particles are more flexible than suggested by rigid cryo-EM or crystal structures. Much research is done on making FMDV VLPs for use in vaccines. The increased knowledge of particle specificity of VHHs can be used for further improving production of VLPs with enhanced immunogenicity.

## Data availability statement

The original contributions presented in the study are included in the article/Supplementary material, further inquiries can be directed to the corresponding author.

## Author contributions

AD, MH, and WP designed and coordinated the study. AD and SS performed grant acquisition and financial reporting. HL and MH performed the experiments. The XL-MS study done at Coval X was coordinated by AD and MH. HL, MH, SS, and AD completed data analysis. MH wrote the first version of the manuscript, that was subsequently edited by HL, AD, and WP. All authors contributed to the article and approved the submitted version.
