# Supplementary material for: Mapping of foot-and-mouth disease virus antigenic sites recognized by single-domain antibodies reveals different 146S particle specific sites and particle flexibility
Source: Front Vet Sci. 2023 Jan 9;9:1040802. doi: 10.3389/fvets.2022.1040802 (PMC9869066; doi:10.3389/fvets.2022.1040802)
Supplement: Supplementary file 1 [file Table_1.DOCX]

| **Supplementary TABLE 1 \|** Coverage of FMDV A24Cru VP1, VP2 and VP3 sequence in peptide mass fingerprints. | | | | | | | |
| --- | --- | --- | --- | --- | --- | --- | --- |
|  | Sequence coverage using different proteases (%) | | | | | |  |
|  | Trypsin | Chymotrypsin | Elastase | ASP-N | Thermolysin | Total |  |
| VP1 | 70.4 | 66.2 | 70.9 | 0 | 0 | 96.7 |  |
| VP2 | 80.7 | 83.9 | 45.4 | 5.0 | 10.1 | 98.6 |  |
| VP3 | 54.3 | 76.5 | 30.3 | 0 | 0 | 97.3 |  |
