# Supplementary material for: Mapping of foot-and-mouth disease virus antigenic sites recognized by single-domain antibodies reveals different 146S particle specific sites and particle flexibility
Source: Front Vet Sci. 2023 Jan 9;9:1040802. doi: 10.3389/fvets.2022.1040802 (PMC9869066; doi:10.3389/fvets.2022.1040802)
Supplement: Supplementary file 2 [file Data_Sheet_1.PDF]

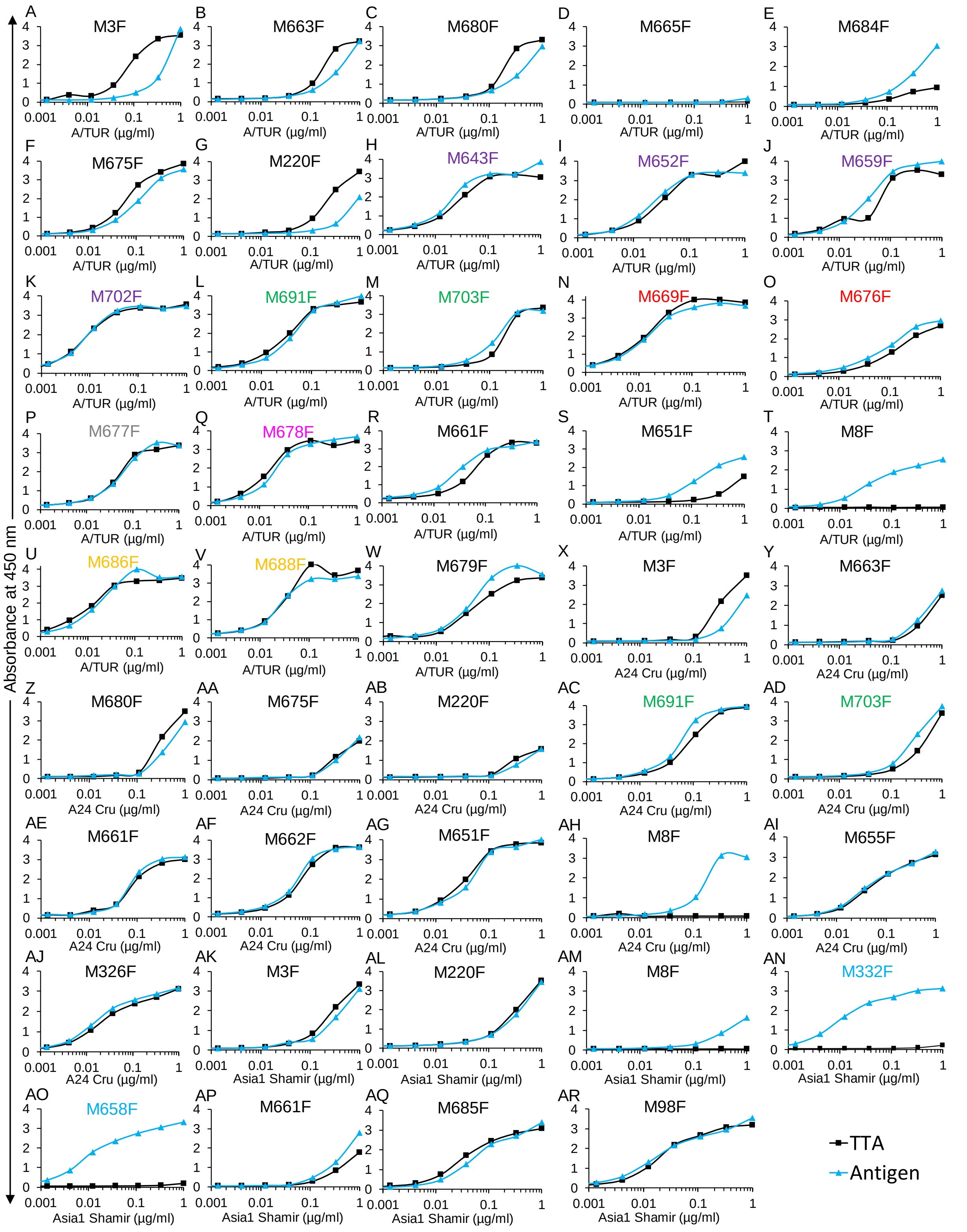

Supplementary FIGURE 1 | Trypsin sensitivity of epitopes recognized by VHHs in ELISA. Untreated FMDV antigen (Antigen) or trypsin-treated antigen (TTA) were titrated in 3-fold dilution series in DAS-ELISAs using the same VHH in unlabeled form for coating and in biotinylated form for detection, as indicated on top of each panel. Each panel (**A-AR**) represents a different combination of VHH and FMDV strain. FMDV strains A/TUR (**A-W**), A24Cru (**X-AJ**) and Asia1 Shamir (**AK-AR**) were used. VHHs are arranged into panels according to their classification into antigenic sites. M655F was deglycosylated before use in ELISA.

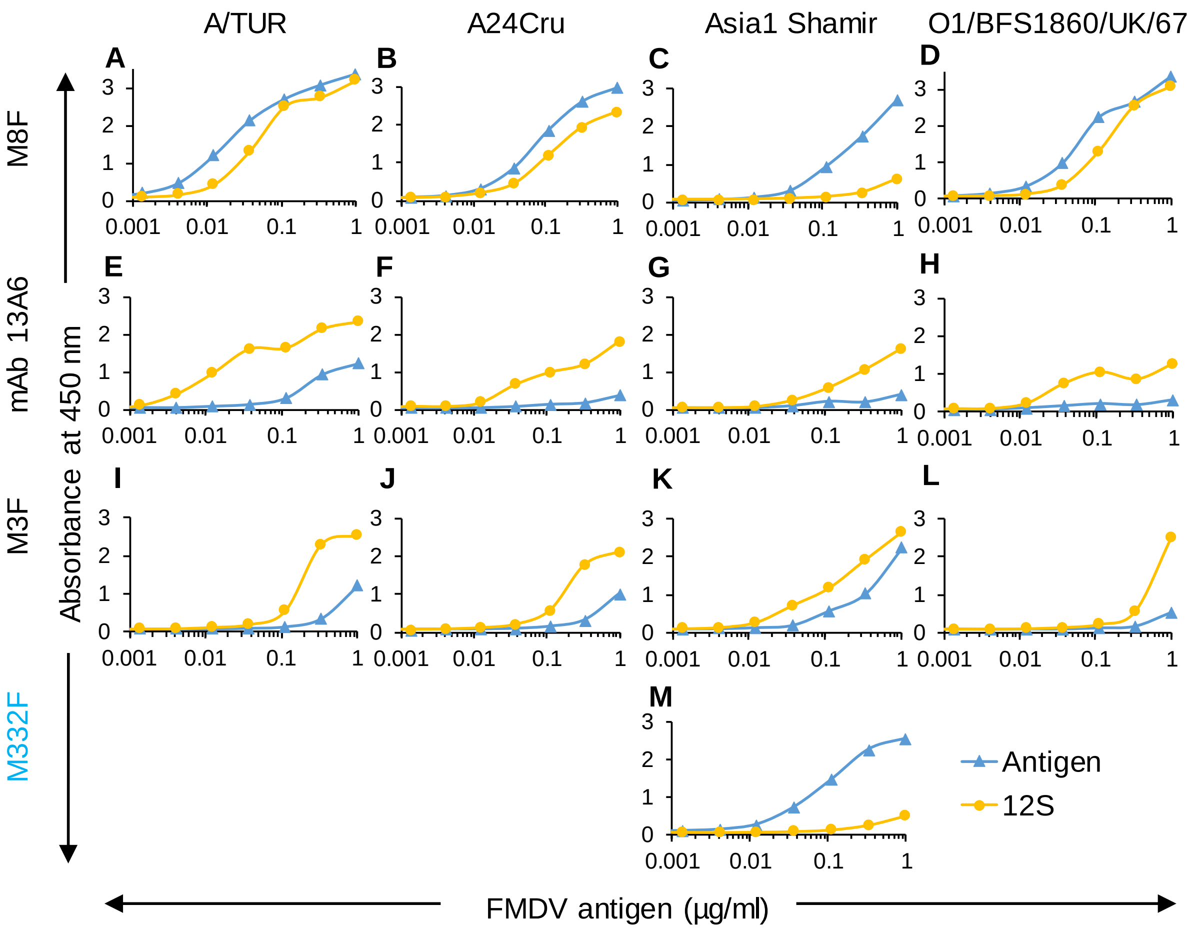

Supplementary FIGURE 2 | Analysis of FMDV particle specificity of various VHHs and mAb 13A6 in ELISA. Untreated FMDV antigen (Antigen) consisting predominantly of 146S particles or antigen that was heated at 56ºC for 1 h (12S) were titrated in threefold dilution series in DAS-ELISAs using the same VHH or mAb 13A6 in unlabeled form for coating and in biotinylated form for detection. Panels **A-M** represent different combinations of VHH or mAb and FMDV strain used, as indicated at the left of panels (VHH or mAb) or above the panels (FMDV strain).

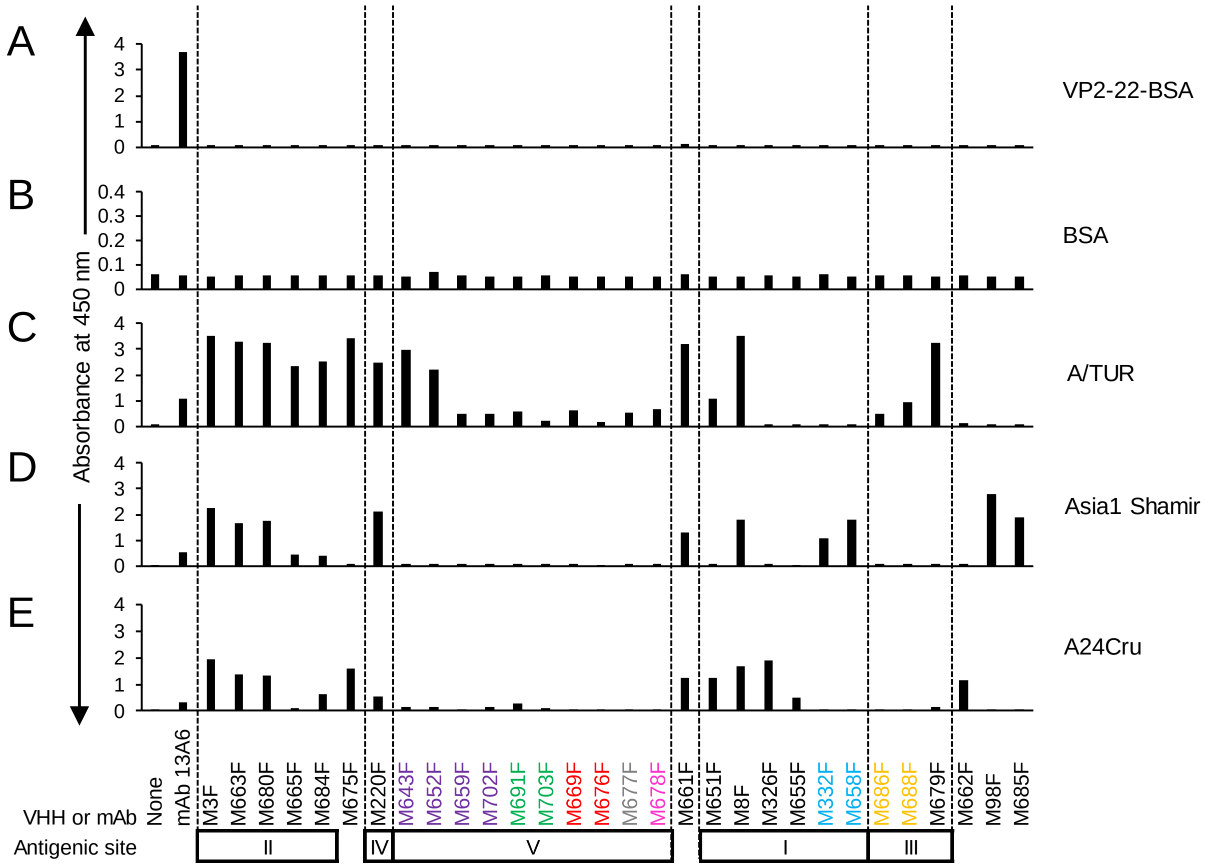

Supplementary FIGURE 3 | VHH binding to a peptide representing the VP2 N-terminus. A peptide representing the N-terminal 22 amino acid residues of VP2 (DKKTEETTLLEDRILTTRNGHT) conjugated to BSA (VP2-22-BSA) was coated to ELISA plates (**A**) and incubated with biotinylated VHHs or biotinylated mAb 13A6 that was detected with streptavidin – HRPO conjugate in ELISA. Controls included plates coated with BSA (**B**) or crude antigen of three FMDV strains (**C-E**) as indicated at the right. Only positive control mAb 13A6 bound the VP2 peptide as shown by the high ELISA signal on VP2-22-BSA but not BSA, while none of the VHHs bound this peptide although they could bind FMDV antigens. VHHs are arranged according to their classification into antigenic sites as shown at the bottom.

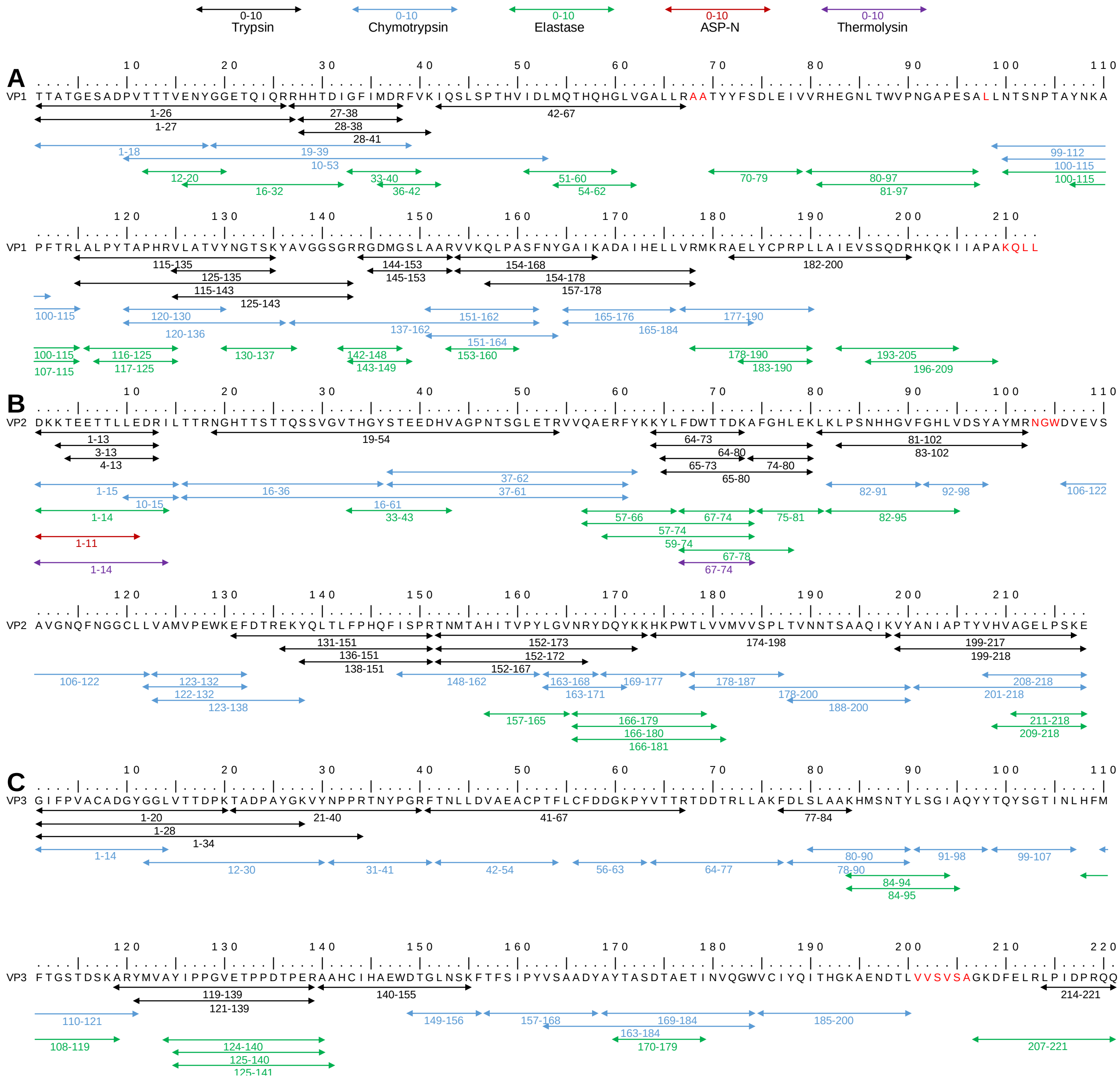

Supplementary FIGURE 4 | Peptide mass fingerprints of FMDV A24Cru viral capsid proteins. Separate peptide mass fingerprints were made for VP1 (**A**), VP2 (**B**) and VP3 (**C**). The peptides identified by digestion with 5 different proteases (see legend at top) are indicated by arrows that are aligned with the A24Cru sequence that was obtained by sequencing of the FMDV strain used. Some amino acid residues (red) are not covered by a peptide.

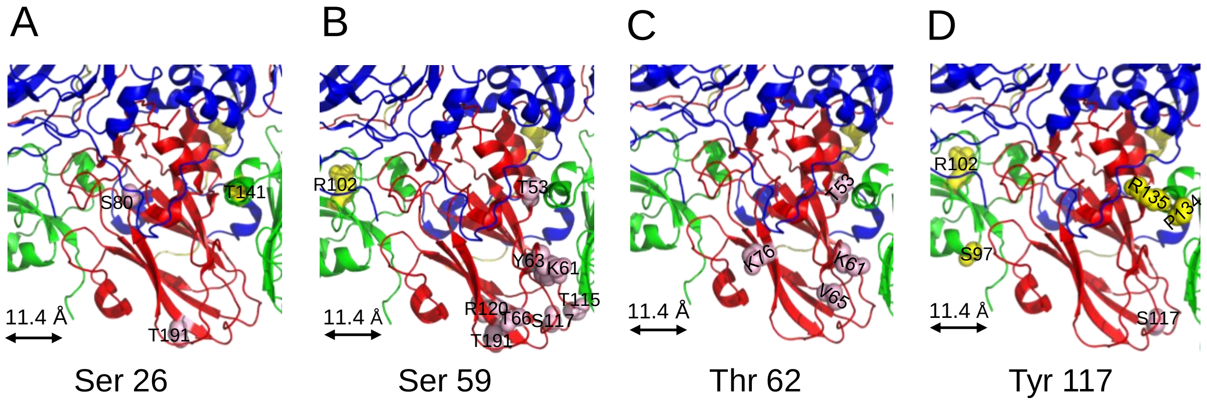

Supplementary FIGURE 5 | Location of FMDV residues that are cross-linked to the same M702F residue. Cross-linked residues are mapped onto the A22IRQ structure (PDB: 4GH4) that is presented as cartoon using PyMOL. Panels **A-D** each show the FMDV residues cross-linked to the M702F residue (IMGT numbering) indicated below each panel. VP1, blue; VP2, green; VP3, red; VP4, yellow. Cross-linked residues are shown with side chains as yellow (VP2) or pink (VP3) spheres. Biheaded arrows indicate the length of the DSS spacer arm (11.4 Å).
